# Applications of the Dixon technique in the evaluation of the musculoskeletal system

**DOI:** 10.1590/0100-3984.2019.0086

**Published:** 2021

**Authors:** Carolina Freitas Lins, Carlos Ernesto Garrido Salmon, Marcello Henrique Nogueira-Barbosa

**Affiliations:** 1 Escola Bahiana de Medicina e Saúde Pública, Salvador, BA, Brazil.; 2 Delfin Inteligência Diagnóstica, Salvador, BA, Brazil.; 3 Departamento de Física, Faculdade de Filosofia, Ciências e Letras de Ribeirão Preto da Universidade de São Paulo (FFCLRP-USP), Ribeirão Preto, SP, Brazil.; 4 Faculdade de Medicina de Ribeirão Preto da Universidade de São Paulo (FMRP-USP), Ribeirão Preto, SP, Brazil.

**Keywords:** Magnetic resonance imaging, Dixon technique, Fat suppression, Fat quantification, Musculoskeletal system, Ressonância magnética, Técnica Dixon, Supressão de gordura, Quantificação de gordura, Sistema musculoesquelético

## Abstract

The acquisition of images with suppression of the fat signal is very useful in clinical practice and can be achieved in a variety of sequences. The Dixon technique, unlike other fat suppression techniques, allows the signal of fat to be suppressed in the postprocessing rather than during acquisition, as well as allowing the visualization of maps showing the distribution of water and fat. This review of the Dixon technique aims to illustrate the basic physical principles, to compare the technique with other magnetic resonance imaging sequences for fat suppression or fat quantification, and to describe its applications in the study of diseases of the musculoskeletal system. Many variants of the Dixon technique have been developed, providing more consistent separation of the fat and water signals, as well as allowing correction for many confounding factors. It allows homogeneous fat suppression, being able to be acquired in combination with several other sequences, as well as with different weightings. The technique also makes it possible to obtain images with and without fat suppression from a single acquisition. In addition, the Dixon technique can be used as a quantitative method, allowing the proportion of tissue fat to be determined, and, in more updated versions, can quantify tissue iron.

## Abstract (pt)

A aquisição de imagens com supressão do sinal da gordura é um recurso de grande utilidade diagnóstica, existindo várias sequências capazes de realizá-la. A técnica Dixon, ao contrário de outras técnicas de supressão de gordura, permite suprimir a contribuição do sinal de gordura no pós-processamento e não durante a aquisição, além de permitir a visualização de mapas com a distribuição da água e da gordura. Esta revisão sobre a técnica Dixon almeja ilustrar os princípios físicos básicos, comparar a técnica com outras sequências de ressonância magnética para supressão ou quantificação de gordura, e descrever suas aplicações no estudo de doenças do sistema musculoesquelético. Muitas variantes da técnica Dixon foram desenvolvidas, proporcionando separação mais consistente dos sinais de gordura e água e permitindo correção de muitos fatores de confusão. Permite obter supressão homogênea de gordura, podendo ser adquirida de forma combinada com várias outras sequências, bem como com diferentes ponderações. Esta técnica possibilita também a obtenção de imagens com e sem supressão de gordura a partir de uma única aquisição. Adicionalmente, a técnica Dixon pode ser utilizada como recurso quantitativo, pois permite a mensuração do porcentual de gordura e, em versões mais atualizadas, consegue quantificar ferro tecidual.

## INTRODUCTION

Magnetic resonance imaging (MRI) is one of the most important and widely used imaging methods in modern medicine, avoiding the use of ionizing radiation. MRI allows the acquisition of images in various anatomical planes, as well as volumetric acquisitions for multiplanar and three-dimensional (3D) analysis^(1)^. In MRI scans, the majority of the signal originates from the nuclei of hydrogen atoms in water and lipid molecules since such molecules are abundant in the human body. For the resonance of those nuclei to occur, a radiofrequency pulse must be applied at the same Larmor frequency as the hydrogen nuclei, which has a linear relationship with the intensity of the magnetic field^(1-3)^.

The pixel intensity in the MRI scan depends on several physical properties of the corresponding voxel in the body, the most important being proton density, resonant frequency, and relaxation times. The precession frequency and longitudinal relaxation time (T1) are the properties that best differentiate water molecules and lipid (fat) molecules on MRI. Because of its short T1, fat tissue shows high signal in most MRI sequences, whereas the water signal has a variable aspect depending on the weighting chosen. The precession frequency and T1 can both be used to generate sequences in which the fat signal can be suppressed, assuming that the contribution of the water signal remains constant^(2)^.

Suppression of the fat signal is a highly useful diagnostic technique that is possible because of the differences between water and fat in terms of the resonant frequencies and T1^(1,2)^. Examples of fat-suppression techniques include frequency-selective saturation pulse sequences, inversion recovery, and hybrid chemical shift-based techniques, such as the Dixon technique^(2,4)^.

The Dixon technique, unlike other fat suppression features, allows the contribution of the fat signal to be suppressed in post-processing rather than during acquisition, as well as providing water and fat distribution maps^(5)^. Several recent studies have demonstrated the application of the Dixon technique for quantitative assessment of the fat fraction (FF), joint cartilage analysis, bone marrow studies, evaluation of sacroiliitis, and magnetic susceptibility artifact reduction in the presence of metallic implants^(2,6-10)^.

In the past, only proton spectroscopy was available for fat-water fraction measurement, but this method has limited spatial resolution compared with imaging techniques. In 1984, Dixon^(11)^ combined the capabilities of spectroscopy and MRI, obtaining signal separation from fat and water to generate, after processing, images with only the water or fat signal, all in one acquisition^(8)^. In images acquired with different echo times, the Dixon technique makes it possible to obtain multiple images based on the chemical shift^(12)^. Variants of this sequence are available from most MRI equipment manufacturers under different acronyms^(13)^: iterative decomposition of water and fat with echo asymmetry and least-squares estimation (IDEAL; GE Healthcare, Waukesha, MI, USA); Dixon (Siemens Healthcare, Erlangen, Germany); mDIXON (Philips Medical Systems, Best, the Netherlands); and FatSep (Hitachi, Tokyo, Japan).

This review of the Dixon technique aims to illustrate the basic physical principles, to compare the technique with other MRI sequences for fat suppression or quantification, and to describe its applications in the study of diseases of the musculoskeletal system, as well as to address its advantages and limitations.

## BASIC PHYSICAL PRINCIPLES

The information that is essential for understanding Dixon techniques is that water and fat are the main components that contribute to forming the images seen on an MRI^(14)^. Hydrogen nuclei have a higher resonant frequency in water molecules than in fat molecules. Within a homogeneous field, the difference in frequency between the main peak of fat molecules and that of water molecules is 3.4 ppm. Therefore, the absolute difference in frequency will change according to the field strength of the equipment, from approximately 217.6 Hz at 1.5 T to approximately 435 Hz at 3.0 T.

**Figure 1:**
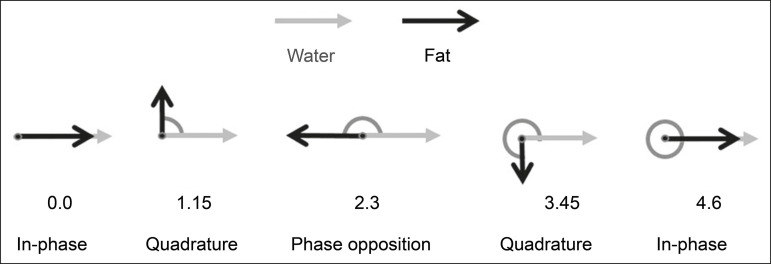
Representation of transverse magnetization vectors of water and fat in acquisitions with different TEs (indicated in ms), based on the resonant frequency of water, in a 1.5 T scanner.

In a gradient-echo (GRE) sequence, transverse magnetization of water and fat begin in-phase immediately after the initial excitation pulse, a condition known as in-phase imaging (Figure 1). After 2.3 ms [= π∕(2*π*217.6)s] in a 1.5 T scanner or 1.15 ms [= π∕(2*π*435)s] in a 3.0 T scanner, these magnetizations will be in opposition, or out-of-phase (180°). If the image is acquired with those echo times (TEs), the water and fat signals are subtracted from each other and can cancel each other out if there is the same amount of water and fat in the voxel (Figure 1). For acquisitions with higher TEs, the water and fat signals can be summed, because they would be in-phase^(5,6)^.

It would be technically impossible to acquire images based on an echo with null TE. Therefore, an acquisition is made at twice the TE of the first out-of-phase condition, the signal containing the sum of both contributions, thus creating the in-phase condition (Figure 1). This is the basic principle of the Dixon technique-image acquisition at two different TEs, known as the two-point Dixon (2PD) technique^(11)^. The 2PD technique is also implemented in a modified spin-echo (SE) pulse sequence, in which the first image corresponds to a conventional SE, with fat and water in-phase, the voxel brightness reflecting the added transverse magnetization of both chemical components, and the second image is acquired with a gradient shifted to produce a 180° phase difference between water and fat.

In the post-processing of the in-phase and out-of-phase images, there is a step for water and fat separation, so that adding the in-phase and out-of-phase images results in a water image, while subtracting the out-of-phase image from the in-phase image gives a fat image^(1)^. Thus, the Dixon technique allows the measurement of the fat-water fraction in a region of interest larger than that achieved with spectroscopy and with higher spatial resolution^(1,13,15,16)^. In general, this concept could be used for the suppression or quantification of fat in various types of pulse sequences^(14)^. 

Some obstacles initially prevented widespread use of the 2PD sequence. First, the magnetic field is not always homogeneous, especially in large areas or regions with air-bone interfaces. Therefore, a phase error, due to heterogeneity of the static (B0) magnetic field, may occur promoting undesired suppression of water and fat signals; in extreme situations, local fat and water signal exchange may occur. If the magnetic field is heterogeneous, the sum and subtraction approach results in incomplete or incorrect separation of water and fat^(5,6,16,17)^.

To address the limitations of the 2PD sequence, the Dixon technique has been modified and evolved to the three-point Dixon technique. Another echo acquisition with a different TE was added to form a system with a single solution of three equations (the three images) and three unknowns (water content, fat content, and heterogeneity), thus making the technique more powerful^(5)^. The three-point Dixon technique provides more effective fat suppression, resulting in a better signal-to-noise ratio (SNR), shorter acquisition time, and optimal spatial resolution^(5,17)^.

The fat signal is generated by a combination of different chemical groups with different chemical shifts (i.e., fat has a multispectral representation). To overcome this difficulty, a combination of image acquisition and processing techniques has been used with multiple acquired echoes^(13)^. However, that requires an algorithm specific for fat and water signal decomposition, which is more complex than simple 2PD subtractions or additions, allowing the efficiency of the signal separation between tissues to be increased^(5,17)^.

Many variants of the Dixon technique have been developed, providing more consistent separation of fat and water signals, as well as allowing correction for many confounding factors^(15)^. The development of the technique has shown that symmetrically acquired echoes can produce artifacts that can degrade image quality. Thus, the Dixon technique became able to combine asymmetrically acquired echoes with an iterative algorithm to maximize noise performance, receiving different designations^(18)^: IDEAL (GE Healthcare), Dixon (Siemens Healthcare), and mDIXON (Philips Medical Systems). This new version of the Dixon technique, using asymmetrically acquired echoes, is highly versatile because it can be applied in T1-, T2-, or proton density-weighted sequences, as well as being able to be combined with SE, fast SE (FSE), balanced steady-state free precession and spoiled gradient-recalled echo sequences, to suppress fat and produce high-quality images with shorter acquisition times^(2,19)^. Another advantage is the manual post-processing quantification of fat for acquisition of the FF, although it is not highly reliable, because of the persistence of some confounding factors^(6,20)^.

Other technological advances have recently been incorporated into the Dixon technique, providing resources for the quantitative analysis of fat and iron content, known as IDEAL-IQ (GE Healthcare); LiverLab (Siemens Healthcare); mDIXON Quant (Philips Medical Systems). These quantitative methods use a more sophisticated fat quantification approach, in which images are acquired at multiple TEs for simultaneous acquisition of FF, water fraction, and transverse relaxation rate (R2*) maps^(21)^.

In some clinical situations, fat and iron deposits overlap in certain organs or tissues, and it is important to measure T2* relaxation time, or its inverse, R2*, allowing quantification of the iron. At sites with iron overload, there is interaction between the iron and water molecules, promoting faster loss of phase coherence, more rapidly reducing transverse magnetization, with a significant decrease in the MRI signal by T2* reduction. Therefore, R2* maps allow a direct correlation with the iron content of the structure analyzed^(22-24)^.

The quantitative Dixon technique is a 3D method of GRE imaging, using magnitude and phase information of an average of six echoes-a six-point Dixon technique-which is appropriate for the multispectral separation of the fat and water signals. In addition, multi-echo acquisition provides vigorous water and fat separation with correction of the T2* effect, and the T1 effect can be avoided by using long repetition times. Thus, the spectral complexity of fat as well as noise can be quite tolerable^(21,25)^. By incorporating an R2* map into the algorithm, the Dixon technique takes the T2* and field inhomogeneity effects into consideration, producing the proton density FF, which does not predispose to a measurement error due to the potential confounding factor of iron overload^(21)^.

## COMPARISON OF OTHER TECHNIQUES FOR FAT SUPPRESSION OR QUANTIFICATION

The Dixon technique has several characteristics that broaden its clinical applications, including homogeneous fat suppression, reduction of magnetic susceptibility artifacts in the presence of metallic materials, and the use after intravenous contrast administration, as well as fat quantification. Of those applications, fat suppression is notable, because it improves the contrast between tissues and highlights certain pathological changes^(5,26,27)^.

**Figure 2:**
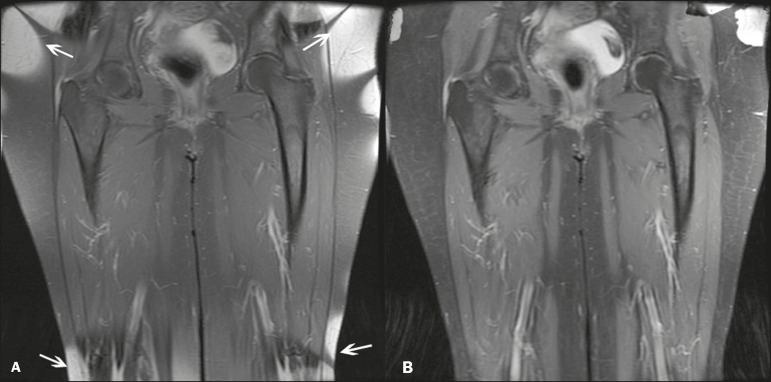
Contrast-enhanced coronal images of the thighs of a 59-year-old female patient with various pulse sequences: T1-weighted FSE sequence with fat suppression (**A**) and T1-weighted IDEAL sequence (**B**). Clear presence of artifacts at the edges of image **A** due to heterogeneity of the B0 or B1 magnetic field when using a wide field of view, impairing fat suppression.

The most common fat suppression techniques are fat selective saturation and short-TI inversion recovery (STIR), both of which are based on the difference between the behavior of water and that of fat in the MRI environment. Since water and fat molecules have different resonant frequencies, this chemical shift allows the use of fat saturation where the saturation radiofrequency pulse has selective frequency centered on the main fat peak, meaning that the total signal will have a minimal fat contribution. Thus, the fat saturation technique can be applied to T1-, T2-, or proton density-weighted images in SE, FSE, or GRE and is reliable for intravenous contrast-enhanced T1 imaging. However, it is prone to incomplete fat suppression, due to the effects of inhomogeneity of the B0 and radiofrequency (B1) magnetic fields, resulting in imperfect fat saturation^(2,4)^, as depicted in Figure 2.

An alternative to the fat saturation technique is the use of STIR sequences, which are based on rapid recovery of the longitudinal magnetization of fat (i.e., its short T1). In STIR sequences, the inversion time is chosen to nullify the contribution of fat after inverting the longitudinal magnetization, being little affected by heterogeneity of the B0 magnetic field. The disadvantages of the STIR sequence include the smaller SNR and the fact that it is not suitable for intravenous contrast-enhanced imaging, because it suppresses the fat signal based on its short T1, and other tissues with a short T1 (such as intravenous contrast-enhanced areas) are therefore also suppressed^(4,5,27)^. In addition, the STIR sequence is affected by the lack of uniformity of the B1 magnetic field, which leads to imperfect inversion in certain regions and ineffective fat suppression.

The choice of the best fat suppression sequence should take into consideration the anatomy of the region analyzed. Therefore, although fat saturation is the sequence most commonly applied in daily practice-because of its fat selectivity, high SNR, and relatively fast examination time at sites where the anatomy presents irregular contours with abrupt changes between soft tissues, bone and metalic implants, as well as in areas requiring a large field of view (spine, thighs, and legs)-the technique promotes artifacts that do not satisfactorily suppress fat^(26-28)^, as shown in Figure 2.

**Figure 3:**
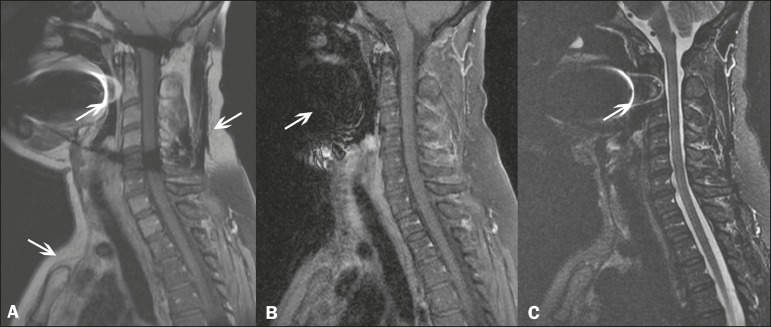
Sagittal images of the cervical spine of a 52-year-old female patient with various pulse sequences: contrast-enhanced T1-weighted FSE with fat suppression (**A**), contrast-enhanced T1-weighted IDEAL (**B**) and STIR (**C**). The IDEAL sequence (**B**) shows a clear reduction of the magnetic susceptibility artifact, especially for visualization of the second cervical vertebra.

**Figure 4:**
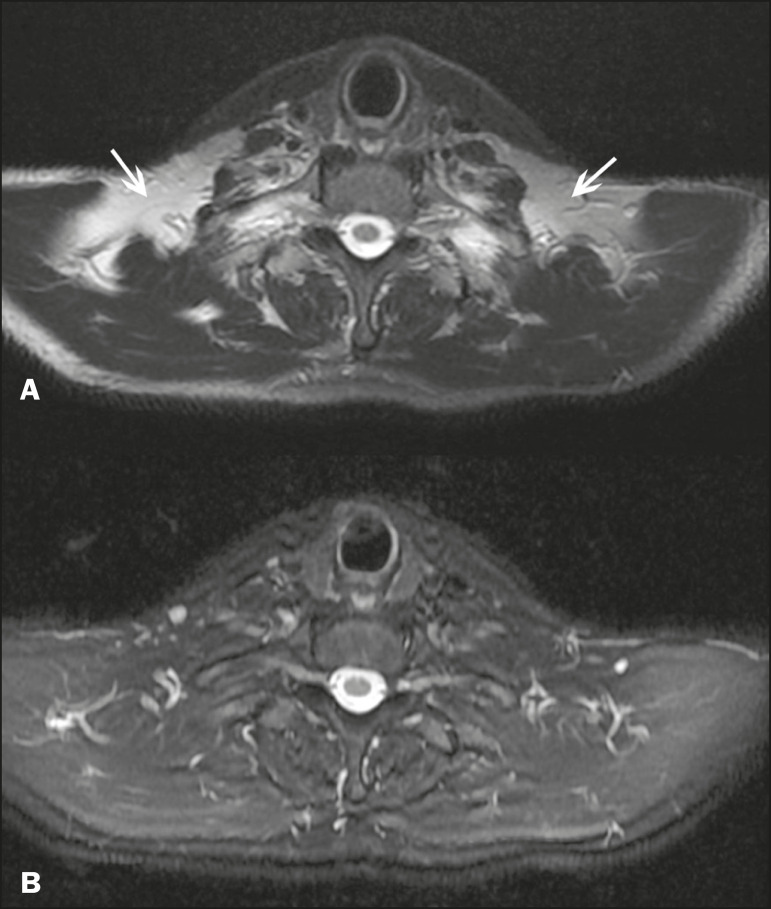
Axial plane images of cervicothoracic transition of a 58-year-old male patient demonstrating insufficient fat suppression in a T2-weighted FSE sequence with fat suppression (**A**), compared with a T2-weighted IDEAL wateronly sequence (**B**).

The multi-point Dixon techniques (with three or more echoes) do not exhibit significant interference by magnetic field heterogeneity and therefore result in efficient fat suppression and are considered by some studies to be better than STIR because they have better SNRs, shorter acquisition times, and equivalent or better spatial resolution^(2,5)^. Therefore, as previously demonstrated^(2,5,27)^, the Dixon technique allows satisfactory images to be obtained in regions with heterogeneity in the local magnetic field (near metallic materials or tissues with excess iron caused by hemosiderosis), as well as in highly susceptible anatomical areas where chemical fat saturation becomes difficult (apex of the neck, hands, feet, and extremities), as depicted in Figures 3 and 4.

In the past, fat was quantified systematically by spectroscopy. However, spectroscopy has an intrinsic limitation to its spatial resolution and requires more complex data processing. In contrast, the Dixon technique has been gaining importance because it allows accurate measurements of the FF, with shorter examination times to cover a large region of interest. However, we should be aware of some confounding factors^(29,30)^: the T1 effect, noise bias, T2* decay, the spectral complexity of fat, and B0 magnetic field heterogeneity.

## THE DIXON TECHNIQUE FOR EVALUATION OF THE MUSCULOSKELETAL SYSTEM

The Dixon technique allows four types of images to be obtained: fat-only, water-only, in-phase, and out-of-phase. It is usually acquired in T1 weighting combined with intravenous contrast administration. In T2 weighting, it can be used for better visualization of the regional anatomy, highlighting the water-only images to detect areas with edema (increased signal). Water-only images represent total fat suppression. Fat-only images, visibly similar to T1-weighted images, allow the study of fat, and it is important to remember that in this case only the fat will have a high signal intensity^(5)^.

### Fat suppression

Fat suppression is a method that is widely used in examinations of the musculoskeletal system, with the objective of improving tissue contrast, thus facilitating the identification of lesions. In most MRI sequences, fat has a high signal intensity. Therefore, for better visualization of areas of edema, it is important to apply fat suppression. However, the acquisition of high-quality MRI images of the musculoskeletal system, with uniform fat suppression, is challenging because it is difficult to obtain magnetic field homogeneity at the extremities^(19)^.

**Figure 5:**
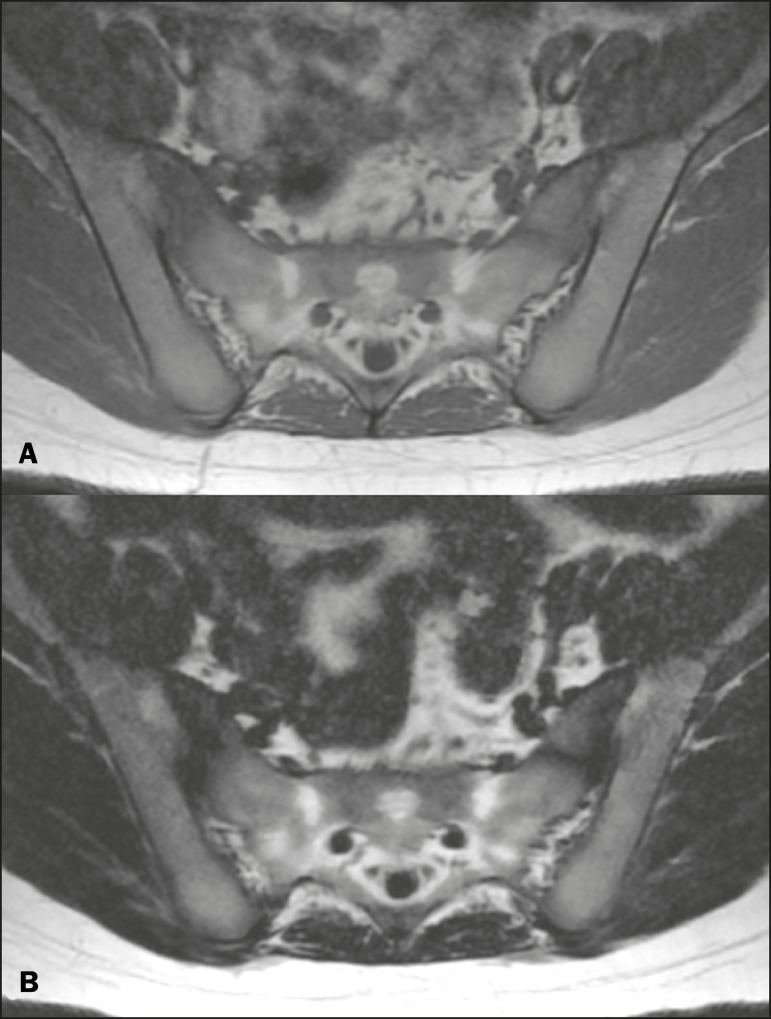
Axial images, from a T1-weighted FSE sequence (**A**) and a T2-weighted IDEAL fat-only sequence (**B**), of a 15-year-old female patient, showing periarticular areas of fatty replacement in the iliac components of both sacroiliac joints, detectable in both sequences.

Because GRE sequences are quite sensitive for detection of metallic artifacts and iron deposition, T2* decay can lead to overestimation those changes, which may limit the evaluation, unlike the Dixon technique^(2)^. Recent studies have attempted to replace T1-weighted sequences with T2-weighted Dixon technique sequences^(8,28)^, using fat-only imaging to detect vertebral metastasis and identify periarticular fatty replacement in cases of sacroiliitis (Figure 5). Those studies obtained similar results in these aspects, demonstrating that the Dixon technique is an interesting option, reducing the duration of the examination, because it would be the only sequence necessary^(8,28,30)^.

The duration of an examination employing the Dixon technique is usually clinically acceptable, albeit about three times longer than the minimum time required to acquire a conventional sequence. In addition, the Dixon technique provides better signal-to-noise efficiency than do conventional fat-suppressed sequences, because the former precludes the need to acquire long spatial spectral pulses, reducing recovery time and compensating for the increased minimum scan time. Thus, the Dixon technique allows effective, homogeneous fat suppression, with a high SNR, aspects that are important for the acquisition of images in the extremities and near metallic implants^(19)^.

### Fat quantification

The use of MRI for the quantification of adipose tissue has been increasing, becoming a subject of great interest and the object of considerable research^(16,31)^. It is most commonly used in some specific areas or organs, such as the liver. There is a need for additional studies to standardize and validate MRI-based fat quantification for clinical application in other tissues, such as muscle and bone marrow^(16)^.

Various studies have addressed the use of fat quantification by the Dixon technique combined with GRE or SE for evaluation of the musculature in diseases such as Duchenne muscular dystrophy^(5,6,13,16)^, as well as of the volume of the rotator cuff muscles^(32-34)^. The quantitative Dixon technique has been used in the analysis of fatty infiltration and muscle volume in patients with a torn rotator cuff, to assist in the indication of surgical repair^(34)^. The advantage of using the Dixon technique in such cases is that it enables volumetric analysis of the ventral surface of the muscle, whereas the Goutallier classification system presents low reproducibility, with evaluation restricted to a single slice^(33)^.

The quantitative Dixon technique methods can also be used in evaluating bone marrow infiltration in hematological disorders, metastatic diseases, and osteoporosis^(15,18,29,30,35)^. Especially in the evaluation of hematological diseases, the technique allows the quantification of fat and of iron deposition, which can accumulate in the body in patients with history of multiple blood transfusions^(6)^. In Gaucher disease, for example, the reduction in the FF may reflect intracellular deposition in the bone marrow. Maas et al.^(35)^ suggested using an FF cutoff point of 0.23, below which there is an increase in the prevalence of complications arising from Gaucher disease of the lumbar spine (vertebral collapse and osteonecrosis). However, that method of quantification warrants further study, including other anatomical regions and other diseases, to improve understanding and to standardize its analysis.

**Figure 6:**
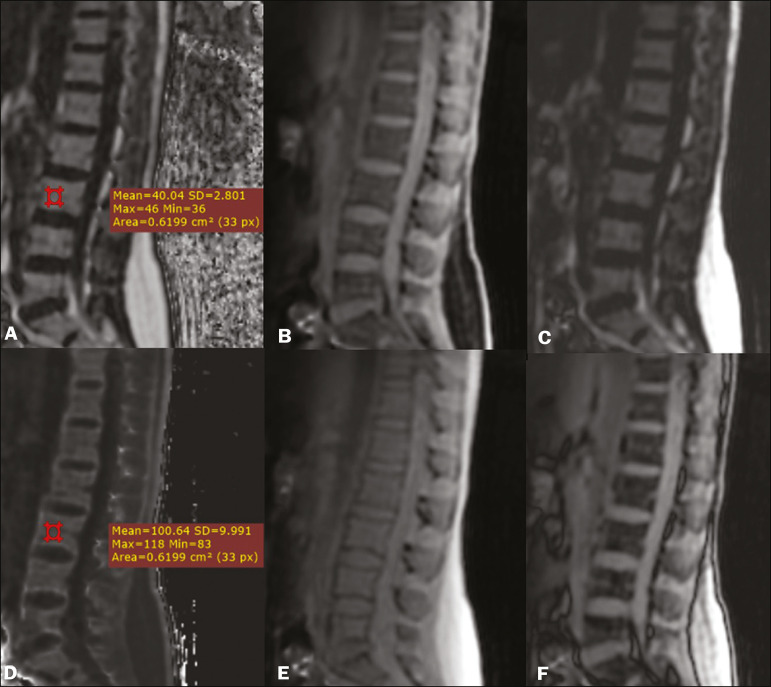
Images obtained in a single acquisition by the modified Dixon technique in a 33-year-old female patient to allow direct quantification of the FF and estimation of iron content. In **A**, the FF map; in **B**, the water-only image; in **C**, the fat-only image; in **D**, the R2* map; in E, the in-phase image; in **F**, the out-of-phase image. Quantitative evaluation with demarcated regions of interest can be illustrated by demonstrating the FF (**A**) and estimating the iron content (**D**).

Quantitative Dixon sequences constitute a promising and reliable method for fat suppression and fat quantification. Through automatic image processing, it provides, in addition to fat-only, water-only, in-phase, and out-of-phase images, FF and R2* images that allow fat quantification and iron content estimation, respectively^(25)^, as shown in Figure 6.

Unfortunately, there are no validated data regarding the best parameters for the acquisition of quantitative Dixon sequences. Therefore, divergences and lack of standardization make it difficult to arrive at universally accepted normal values for the measurements obtained by these sequences^(13,16)^. When the IDEAL-IQ, LiverLab, and mDIXON Quant methods are employed, even greater care should be taken in the interpretation of information related to the musculoskeletal system, because the technique is more well developed and more widely used for evaluation of the liver^(21)^.

Sequences obtained with the quantitative Dixon technique are becoming available for various makes of MRI scanners. The acquisition time is variable, being longer (typically 4-5 min) for the IDEAL, Dixon, and mDIXON sequences, whereas it is considerably shorter (16-70 s) for the IDEAL-IQ, LiverLab, and mDIXON Quant sequences, and the images can be acquired during a breath hold or during free breathing, depending on the region analyzed. Because the acquisition time is relatively long for bone marrow evaluation, it can be performed in free breathing. It is also important to emphasize that the images obtained in the IDEAL, Dixon or mDIXON sequences have higher spatial resolution, allowing better identification of the anatomical structures. The IDEAL-IQ, LiverLab, and mDIXON Quant prioritize quantification and therefore lose a little in terms of anatomical resolution.

In practice, the procedure for the quantification of fat and iron using quantitative Dixon techniques is simple. The MRI scanner itself processes the images, providing all of the maps needed for direct measurement. The evaluator will need only to access the workstation and select the FF or R2* maps through a volume viewer. When the region of interest tool is activated with a shape and size appropriate to the anatomical region studied (Figure 6), the mean value for the FF and the estimated iron content in that area appear^(15,21)^. It is important to mention that R2* is the inverse of T2*, the latter being used in order to measure the amount of iron, a low T2* value indicating a high iron content, which results in a high proton relaxation rate. Therefore, a higher R2* value translates to a lower T2* value and a greater amount of iron deposited in the region of interest analyzed^(22)^.

In clinical practice, the Dixon technique has great utility and potential in the evaluation of the musculoskeletal system, not only as a robust, efficient fat suppression technique but also for quantitative analysis. Table 1 summarizes the main applications of the Dixon technique in the musculoskeletal system.

**Table 1:** Main applications of the Dixon technique in musculoskeletal diseases.

Fat suppression	Fat quantification
• Examination of the hands or fingers, where the transition between soft tissues and ambient air occurs abruptly due to the small dimensions of the structures analyzed (Figure 7) • In the assessment of the pelvis, thighs, legs, and spine, where fat suppression can be more homogeneous, especially in the extremities, even in the presence of metal prosthe-ses (Figures 2 and 3) • In studies of the brachial plexus study because of the potentially higher SNR and larger matrix, allowing larger fields of vision with higher spatial resolution (Figure 4) • Analysis of sacroiliac joints better than that of conventional sequences for subchondral fatty replacement, periarticular bone edema, and bone erosion in a single acquisition (Figure 5) • Identification of small subchondral fracture lines and osteochondral lesions not identified with conventional sequences (Figure 8)	• Quantitative assessment of muscle tropism by the degree of fatty replacement • Quantification of bone marrow fat and cellularity in some hematological disorders, metastatic diseases, and osteoporosis

**Figure 7:**
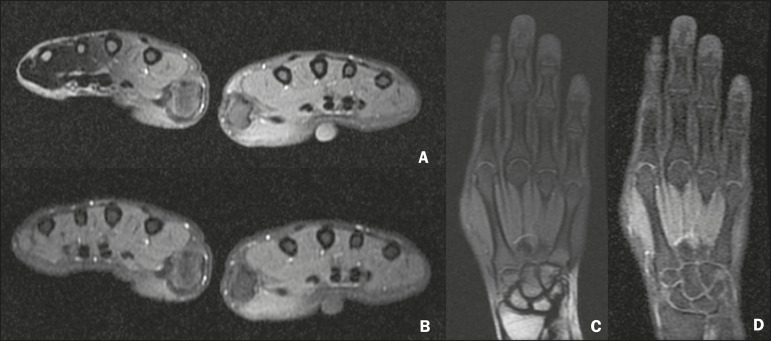
Short- and long-axis images of the hands and wrists of a 30-year-old female patient with psoriatic arthritis followed by a protocol for rheumatologic disease with simultaneous contrast-enhanced imaging of both hands. Contrast-enhanced T1-weighted FSE sequence with fat suppression (**A,C**) and contrastenhanced T1-weighted IDEAL sequence (**B,D**), demonstrating that the latter (the Dixon technique) provided more effective fat suppression in the hand and wrist.

**Figure 8:**
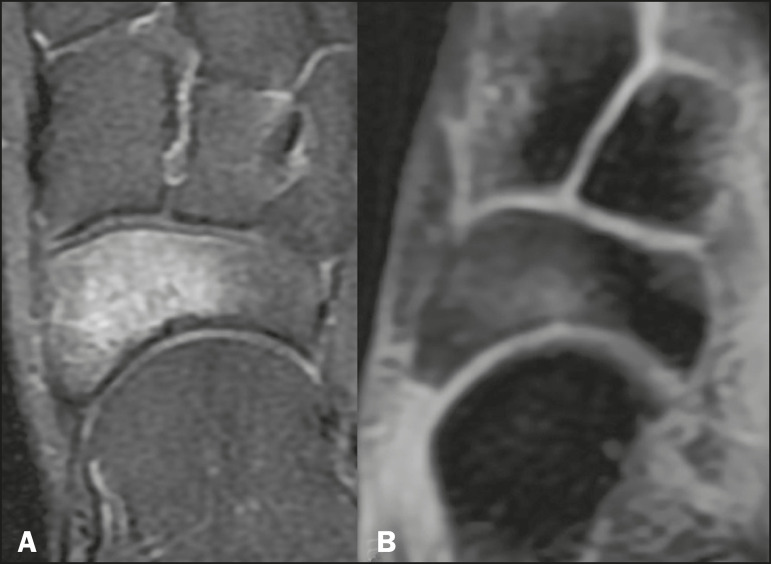
T2-weighted FSE sequence with fat suppression (**A**) and IDEAL water-only sequence (**B**) images of a 24-year-old female patient showing na osteochondral lesion in the navicular bone. The Dixon technique provides better delineation of chondral involvement, whereas bone marrow edema is more evident in the T2-weighted FSE sequence with fat suppression.

## DISADVANTAGES AND LIMITATIONS

As previously mentioned, the Dixon technique has many positive aspects and has been refined to minimize its potential disadvantages. Currently, the main restrictions on its use are that it is not available on less modern equipment and that knowledge regarding its applications is not widely disseminated, as well as the longer acquisition times and the longer time required for image reconstruction when compared with other fat suppression sequences^(1,5)^.

The Dixon technique is not recommended for fat quantification in regions highly affected by heterogeneity of B0 magnetic field, reducing the accuracy of measurements. Therefore, imaging of extremely large regions should be avoided, since the homogeneity correction system of the MRI equipment might not be sufficient for accurate quantification. Despite the limited spatial resolution of spectroscopy, it has higher frequency resolution than the Dixon technique. Dixon technique is not suitable to discriminate between different lipid molecules.

## CONCLUSION

The Dixon technique has advantages in multiple applications for evaluation of musculoskeletal system diseases. It allows more robust fat suppression than do other sequences and can be used in combination with multiple different sequences (GRE and SE) and using different weightings (T1, T2, or proton density). The Dixon technique also allows images with and without fat suppression to be obtained in a single acquisition, as well as being a useful quantitative method, allowing the FF measurement and, in more updated versions, quantifying tissue iron content.

## References

[1] Berglund J (2011). Separation of water and fat signal in magnetic resonance imaging: advances in methods based on chemical shift.

[2] Pezeshk P, Alian A, Chhabra A (2017). Role of chemical shift and Dixon based techniques in musculoskeletal MR imaging. Eur J Radiol.

[3] Westbrook C, Roth CK, Talbot J (2013). Ressonância magnética: aplicações práticas.

[4] Brandão S, Seixas D, Ayres-Basto M (2013). Comparing T1-weighted and T2-weighted three-point Dixon technique with conventional T1-weighted fat-saturation and short-tau inversion recovery (STIR) techniques for the study of the lumbar spine in a short-bore MRI machine. Clin Radiol.

[5] Guerini H, Omoumi P, Guichoux F (2015). Fat suppression with Dixon techniques in musculoskeletal magnetic resonance imaging: a pictorial review. Semin Musculoskeletal Radiol.

[6] Burakiewicz J, Sinclair CDJ, Fischer D (2017). Quantifying fat replacement of muscle by quantitative MRI in muscular dystrophy. J Neurol.

[7] Bredella MA, Losasso C, Moelleken SC (2001). Three-point Dixon chemical-shift imaging for evaluating articular cartilage defects in the knee joint on a low-field-strength open magnet. AJR Am J Roentgenol.

[8] Özgen A (2017). The value of the T2-weighted multipoint Dixon sequence in MRI of sacroiliac joints for the diagnosis of active and chronic sacroiliitis. AJR Am J Roentgenol.

[9] Kijowski R, Tuite M, Passov L (2008). Cartilage imaging at 3.0T with gradient refocused acquisition in the steady-state (GRASS) and IDEAL fat-water separation. J Magn Reson Imaging.

[10] Park EH, Lee KB (2017). Usefulness of black boundary artifact on opposed-phase imaging from turbo spin-echo two-point mDixon MRI for delineation of an arthroscopically confirmed small fracture of the lateral talar dome: a case report. Medicine (Baltimore).

[11] Dixon WT (1984). Simple proton spectroscopic imaging. Radiology.

[12] Berglund J, Ahlström H, Johansson L (2011). Two-point dixon method with flexible echo times. Magn Reson Med.

[13] Noble JJ, Keevil SF, Totman J (2014). In vitro and in vivo comparison of two-, three- and four-point Dixon techniques for clinical intramuscular fat quantification at 3 T. Br J Radiol.

[14] Ma J (2008). Dixon techniques for water and fat imaging. J Magn Reson Imaging.

[15] Yoo HJ, Hong SH, Kim DH (2017). Measurement of fat content in vertebral marrow using a modified dixon sequence to differentiate benign from malignant processes. J Magn Reson Imaging.

[16] Grimm A, Meyer H, Nickel MD (2018). Evaluation of 2-point, 3-point, and 6-point Dixon magnetic resonance imaging with flexible echo timing for muscle fat quantification. Eur J Radiol.

[17] Del Grande F, Santini F, Herzka DA (2014). Fat-suppression techniques for 3-T MR imaging of the musculoskeletal system. Radiographics.

[18] Takasu M, Kaichi Y, Tani C (2015). Iterative decomposition of water and fat with echo asymmetry and least-squares estimation (IDEAL) magnetic resonance imaging as a biomarker for symptomatic multiple myeloma. PloS One.

[19] Gerdes CM, Kijowski R, Reeder SB (2007). IDEAL imaging of the musculoskeletal system: robust water fat separation for uniform fat suppression, marrow evaluation, and cartilage imaging. AJR Am J Roentgenol.

[20] Reeder SB, Pineda AR, Wen Z (2005). Iterative decomposition of water and fat with echo asymmetry and least-squares estimation (IDEAL): application with fast spin-echo imaging. Magn Reson Med.

[21] Eskreis-Winkler S, Corrias G, Monti S (2018). IDEAL-IQ in an oncologic population: meeting the challenge of concomitant liver fat and liver iron. Cancer Imaging.

[22] Chavhan GB, Babyn PS, Thomas B (2009). Principles, techniques, and applications of T2*-based MR imaging and its special applications. Radiographics.

[23] Wood JC, Enriquez C, Ghugre N (2005). MRI R2 and R2* mapping accurately estimates hepatic iron concentration in transfusion-dependent thalassemia and sickle cell disease patients. Blood.

[24] Verlhac S, Morel M, Bernaudin F (2015). Liver iron overload assessment by MRI R2* relaxometry in highly transfused pediatric patients: an agreement and reproducibility study. Diagn Interv Imaging.

[25] Wang Q, Ye F, Ma P (2019). Quantitative magnetic resonance imaging evaluation of hepatic fat content with iron deposition: will it be disturbed?. J Int Med Res.

[26] Aoki T, Yamashita Y, Oki H (2013). Iterative decomposition of water and fat with echo asymmetry and least-squares estimation (IDEAL) of the wrist and finger at 3T: comparison with chemical shift selective fat suppression images. J Magn Reson Imaging.

[27] Kirchgesner T, Perlepe V, Michoux N (2018). Fat suppression at three-dimensional T1-weighted MR imaging of the hands: Dixon method versus CHESS technique. Diagn Interv Imaging.

[28] Maeder Y, Dunet V, Richard R (2018). Bone marrow metastases: T2-weighted Dixon spin-echo fat images can replace T1-weighted spin-echo images. Radiology.

[29] Li GW, Xu Z, Chen QW (2014). Quantitative evaluation of vertebral marrow adipose tissue in postmenopausal female using MRI chemical shift-based water-fat separation. Clin Radiol.

[30] Shen W, Gong X, Weiss J (2013). Comparison among T1-weighted magnetic resonance imaging, modified dixon method, and magnetic resonance spectroscopy in measuring bone marrow fat. J Obes.

[31] Gee CS, Nguyen JT, Marquez CJ (2015). Validation of bone marrow fat quantification in the presence of trabecular bone using MRI. J Magn Reson Imaging.

[32] Davis DL, Kesler T, Gilotra MN (2019). Quantification of shoulder muscle intramuscular fatty infiltration on T1-weighted MRI: a viable alternative to the Goutallier classification system. Skeletal Radiol..

[33] Matsumura N, Oguro S, Okuda S (2017). Quantitative assessment of fatty infiltration and muscle volume of the rotator cuff muscles using 3-dimensional 2-point Dixon magnetic resonance imaging. J Shoulder Elbow Surg.

[34] Micevych PS, Garg A, Buchler LT (2019). Optimizing methods to quantify intramuscular fat in rotator cuff tears with normalization. Skeletal Radiol.

[35] Maas M, van Kuijk C, Stoker J (2003). Quantification of bone involvement in Gaucher disease: MR imaging bone marrow burden score as an alternative to Dixon quantitative chemical shift MR imaging-initial experience. Radiology.

